# Annotations

**Published:** 1895-10-19

**Authors:** 


### Annotations.

### Large or Small Families ?

That the question whether a large or a small family is desirable has long passed beyond the stage of mere speculation is amply proved by certain statistics recently collected by M. de Foville, and quoted by Mr. Benjamin Kidd in his "Social Evolution." According to those statistics the birth-rate in most European countries has, in twenty years, fallen in a very remarkable degree. At the beginning of the period in question the birth-rate in Prussia was 39·1 per 1,000 of the population, at the end it was 36·3 ; in Switzerland the rate was 35·5 at the beginning, and 32·5 at the end; in England the period opened with a birth-rate of 35·5, and closed with one of 33·7 ; in Scotland and Ireland the rate was 24·9 at the commencement, and 23·6 at the close. Similar falls have been noted in all other European countries, with the exception of Austria and Hungary. It is not difficult to explain why this condition of things should have come about. But the question for consideration is rather the "whither" than the "wherefore." Whither does this tendency to the avoidance of marriage and of large families lead? It leads, necessarily, to a relative lowering of population; and that may be an advantage. But in France it is said to lead in another direction, which is not only not advantageous, but is disastrous. In that country the average family consists of not more than one or two children. One consequence of this is an excessive tenderness on the part of parents, and perpetual fears of misfortune happening to their offspring. The latter are excessively indulged in childhood, and so guarded and protected against every possibility of harm, that the male children tend to lose all boldness, and grow up almost entirely destitute of enterprise and power of endurance. No robust-minded person can fail to see that here is an evil of a fatal character. If the children of a country are in detail without pluck or enterprise, it is certain that the whole adult nation will soon be in the same condition. This is an object lesson which English men and women will do well to lay to heart. If our excessive dread of small means for ourselves and our offspring tends directly to the ruin of the nation's boldness and enterprise, the outlook may well give us pause.

### The Health of Shanghai.

Everything Chinese has a special interest for Europeans at the present moment. Shanghai is eminently Chinese, and yet, for China, eminently European. A geographical scholar was asked the other day how many foreigners—that is, European and other persons foreign to China—he supposed there were in Shanghai. He opined there might be a thousand. As a matter of fact, there are five thousand, and half the five thousand are British. The health of Shanghai, and the provisions for preserving its health, are, therefore, matters of great importance to a good many thousands in this country. The Chinese portion of Shanghai is nearly as large as Liverpool or Glasgow, and it consists of narrow, unwholesome streets, filthy dwellings, and every insanitary abomination for which so many Asiatic cities are notorious. So much for the vaunted intelligence and capacity of the Confucians. The Europeans all live together in the European quarter; and there they have broad, well-lighted streets, healthy houses, and, as far as possible, all the resources of European sanitary science. As showing how much Shanghai now resembles the London or Paris of the middle ages, it may be noted that only last year the city was threatened with an epidemic of the Black, or Bubonic Plague, the very plague which nearly decimated London in the seventeenth century. In such circumstances the European doctors were the first to be consulted by the Chinese authorities. Every conceivable scientific measure of prevention was employed, and, fortunately, no entrance was effected by the dread visitant, in spite of the fact that great ravages had been made at Pakhoi, Canton, and Hong Kong. The British and other foreign residents of Shanghai have had the true British good sense to provide themselves with a medical officer of health, and he is an Englishman. Dr. Henderson's annual reports for 1893 and 1894 are now before us. The first thing one naturally looks for is the death rate of the city, or rather of the British or non-Chinese portion of it. That death rate compares favourably with the death rate of Liverpool or Hull, similar seaport towns. Moreover, there is shown between 1870 and 1893 an almost continuous decline, the rate standing at 28·6 per thousand for the former year, and at 18·4 for the latter. This is a striking object lesson, and is, no doubt, entirely due to the adoption of the methods of European sanitary science. The disease rate, like the death rate, is low, and zymotic diseases in the European quarter are pleasingly few. Altogether, these annual reports of Dr. Henderson show that where British men and British medical science go, there go common sense, practical efficiency, and wholesome living ; with those rewards of sound health and almost entire freedom from the ghastly terrors of unknown and dreaded epidemics, which haunt like a nightmare the imagination of Oriental peoples.

### "The Current that Kills."

Many people have as genuine a terror of electricity as their ancestors had of ghosts and churchyards at midnight. This is quite foolish. As in the case of ghosts, it is ignorance which frightens, but knowledge which reassures. Dr. J. B. Verity, in his "Electricity up to Date," tells us that though "some fatal results have happened, electricity does not kill wholesale, as does dynamite, gunpowder, or coal-gas explosions; nor is the list of fatal accidents to be compared with deaths due to exploding or overturned oil lamps." It is well to remember that the ability to bear electric currents varies in different individuals. Moreover, that electricity will certainly kill if contact be good and pressure sufficient, need not be denied. Indeed, it will kill very promptly and certainly; so much so, that Dr. Verity thinks capital punishment by electrocution is much the most speedy and painless of all the methods of killing known to science. On this point he reminds us that, since electricity travels much quicker than sensory nerve currents, the brain is rapidly rendered insensitive and the subject therefore becomes quite unconscious long before the sensory nerves can communicate the impression of pain." "The current that kills" is, however, as perfectly under the control of those who have but a little real knowledge as is the gas burner or even the composite candle.

